# Protein Distributions from a Stochastic Model of the *lac* Operon of *E. coli* with DNA Looping: Analytical solution and comparison with experiments

**DOI:** 10.1371/journal.pone.0102580

**Published:** 2014-07-23

**Authors:** Krishna Choudhary, Stefan Oehler, Atul Narang

**Affiliations:** 1 Department of Biochemical Engineering & Biotechnology, Indian Institute of Technology, Delhi, India; Imperial College London, United Kingdom

## Abstract

Although noisy gene expression is widely accepted, its mechanisms are subjects of debate, stimulated largely by single-molecule experiments. This work is concerned with one such study, in which Choi et al., 2008, obtained real-time data and distributions of Lac permease in *E. coli*. They observed small and large protein bursts in strains with and without auxiliary operators. They also estimated the size and frequency of these bursts, but these were based on a stochastic model of a constitutive promoter. Here, we formulate and solve a stochastic model accounting for the existence of auxiliary operators and DNA loops. We find that DNA loop formation is so fast that small bursts are averaged out, making it impossible to extract their size and frequency from the data. In contrast, we can extract not only the size and frequency of the large bursts, but also the fraction of proteins derived from them. Finally, the proteins follow not the negative binomial distribution, but a mixture of two distributions, which reflect the existence of proteins derived from small and large bursts.

## Introduction

Data from many independent experiments show that the abundance of any given protein varies among individual cells of isogenic populations growing under identical conditions [1]–[3]. Early experiments with fluorescent reporters showed that such non-uniformity in protein abundance was due to the inherent stochasticity of gene expression (intrinsic noise) and various forms of cell-to-cell variation (extrinsic noise) [4], [5]. The subsequent development of single-molecule techniques has led to deeper insights into the molecular mechanisms generating the noise [6], [7]. By measuring the number of mRNAs in single cells, Golding et al. showed that transcription was too bursty to be modeled as a Poisson process [8]. Cai et al. [9] and Yu et al. [10] developed two different methods for measuring the number of proteins in single cells. The real-time data of both studies showed that protein synthesis was bursty, and the burst size was exponentially distributed. Under this condition, the steady state protein distribution follows the Gamma distribution, 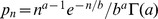, where 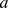 and 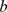 denote the mean burst frequency and burst size [11]. Cai et al. and Yu et al. showed that the Gamma distribution could fit their steady state data, and the values of the mean burst frequency and size derived from the steady state data agreed well with those obtained from real-time measurements.

**Figure 1:**
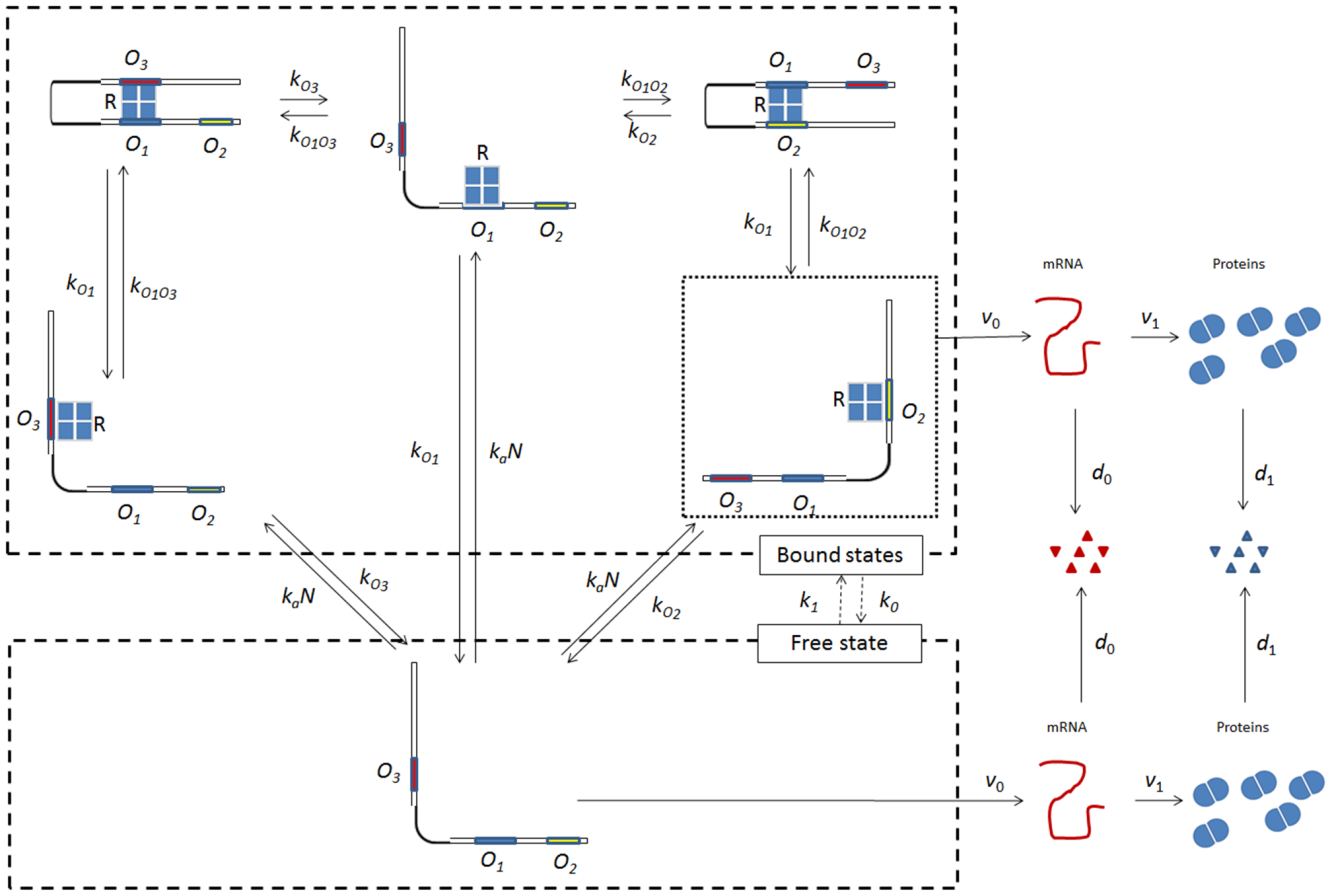
Structure and states of the *lac* operon. The repressor 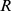 can bind to any of the three operators, namely the main operator 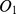, and the two auxiliary operators 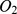, 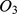. The repressor-free state is enclosed by the lower dashed box. The repressor-bound states, enclosed by the upper dashed box, consist of the following 5 states (clockwise from the left): the 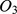 -bound state 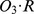, the looped state 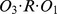, the 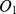 -bound state 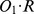, the looped state, 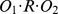, and the 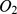-bound state 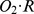. Transcription occurs only if the operon is in the repressor-free state or the repressor-bound state 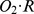. Small bursts occur whenever the repressor dissociates from the looped state 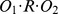 to form the 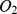 -bound state 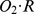. Large bursts occur whenever the repressor dissociates from the DNA to form the repressor-free state. Transitions between repressor-free and repressor-bound states occur with propensities 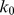 and 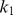.

Armed with these results, Choi et al. [12] attacked a long-standing problem. When non-induced cells of *E. coli* are exposed to small concentrations of the gratuitous inducer TMG, the *lac* operon is induced by stochastic switching of individual cells from the non-induced to the induced state [13]. Choi et al. sought the molecular mechanism of this stochastic switching. To this end, they first quantified the minimum number of LacY molecules required to switch a cell to the induced state, and found this threshold to be 375 molecules. They then suggested a molecular mechanism capable of yielding this threshold by appealing to the known mechanisms of repression and transcription of the *lac* operon. Repression is mediated by the stable DNA loops formed when the Lac repressor is simultaneously bound to the main and auxiliary operators (Fig. 1). Transcription can take place either due to *partial dissociations*, which occur when a repressor trapped in a DNA loop dissociates from the main operator, but not the auxiliary operator; or *complete dissociations*, which occur when the repressor dissociates completely from the DNA. Choi et al. hypothesized that since a partially dissociated repressor remains attached to the DNA, it rapidly rebinds to the main operator, thus limiting the number of transcription events. Although the evidence suggests that no more than one mRNA is made during a partial dissociation, it is conceivable that multiple transcripts are made during a partial dissociation despite its short lifetime, thus leading to a *small transcriptional burst*. In contrast, a completely dissociated repressor takes a relatively long time to find an operator, which results in a *large transcriptional burst*. These large transcriptional bursts can provide enough proteins to cross the threshold for stochastic switching.

**Figure 2:**
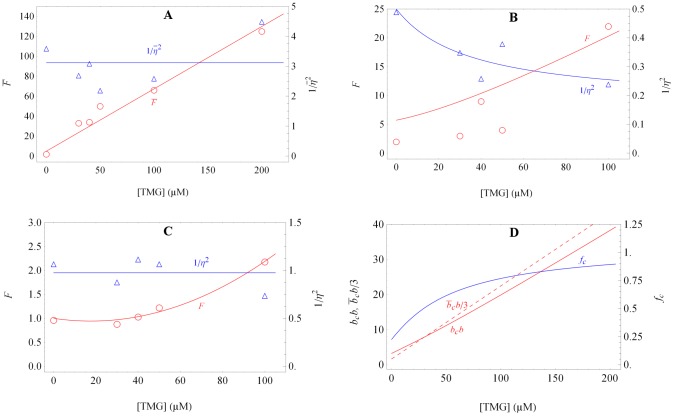
The variation of the Fano factor and the reciprocal of the noise with the inducer level [12]. (a) Derived from data for strain SX703, which exhibits only large transcriptional bursts, since it lacks both auxiliary operators. Choi et al. proposed that 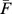 and 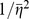 represent the size and frequency of large transcriptional bursts. (b) Derived from raw data for strain SX701, which exhibits mostly small transcriptional bursts, since it has both auxiliary operators. Choi et al. did not consider this data on the grounds that the occurrence of large bursts in a few cells distorted the statistics of the small transcriptional bursts. (c) Derived from data for strain SX701 that was filtered by rejecting the data corresponding to the few cells exhibiting large bursts. Choi et al. proposed that this 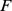 and 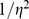 represent the size and frequency of small transcriptional bursts. (d) Mean size of large transcriptional bursts in strain SX701, 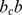, (full red curve) and fraction of proteins derived from such bursts, 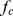, (full blue curve) estimated from the data in (b). The ordinate of the dashed red line is one-third of the ordinate of the 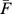 vs. [TMG] line shown in (a), and therefore represents one-third of the (large) transcriptional burst size in strain SX703. The proximity of the full and dashed red lines implies that the mean size of large transcriptional bursts in strain SX701 is approximately one-third of the transcriptional burst size in strain SX703, which is consistent with our model predictions.

Choi et al. tested the foregoing hypotheses as follows. The statistics of small transcriptional bursts were obtained with strain SX701, a 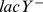 strain that exhibits mostly small bursts. To capture the statistics of large bursts, they deleted the auxiliary operators of their 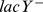 cells, thus creating strain SX703 which yields only large bursts. The statistics of the small and large bursts were quantified by measuring the steady-state protein distributions for both strains at various inducer concentrations. They then concluded, based on the model of Friedman et al. [11], that if 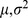 denote the mean and variance of a protein distribution obtained with strain SX701, then the Fano factor, 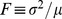, and the reciprocal of the noise, 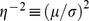, represent the size and frequency of the small bursts. Likewise, if 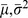 denote the mean and variance for SX703, then 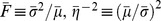 represent the size and frequency of the large bursts. Analysis of the data for SX703 with this method showed that 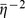 did not change with inducer levels, but 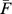 increased dramatically (Fig. 2a), thus confirming their hypothesis that large bursts can generate enough proteins to trigger stochastic switching. Surprisingly, analysis of the data for SX701 also yielded similar trends (Fig. 2b), but this was attributed to the distortions created by the few cells exhibiting large bursts. Indeed, if the data were filtered by removing the contribution of large bursts, 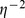 and 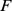 did not change much with the inducer concentration (Fig. 2c), leading the authors to conclude that the small burst frequency and size were independent of the inducer level.

Choi et al. also explained these results by appealing to the known states of the *lac* operon (Fig. 1). However, the mathematical model of Friedman et al., which forms the basis of their data analysis, does not account for these complexities — it only considers a constitutive (unregulated) promoter. Consequently, there is no strong support for the assumption that the proteins follow the Gamma distribution; 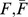 represent the size of small and large bursts; and 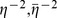 represent the frequency of small and large bursts. The goal of this study is to verify the validity of these assumptions by formulating a stochastic model accounting for the known states of the operon, and deriving analytical expressions for the steady state protein distribution, Fano factor, and noise.

There are stochastic models accounting for the details shown in Fig. 1 [14]–[16], but these studies do not give analytical expressions for the steady state protein distribution. The literature also contains several stochastic models of gene regulation for which analytical solutions were obtained [11], [17]–[24], but they do not account for the presence of multiple auxiliary operators and DNA looping. Our model fills this gap in the theoretical literature, and its analysis yields deeper insights into the experimental data. Specifically, we show that the size and frequency of small bursts cannot be extracted from the data for strain SX701 because they are averaged out. However, we can extract not only the size and frequency of the large bursts, but also their contribution to total protein synthesis, provided the data is not filtered (Fig. 2d). This result also yields tests for the consistency of the model by providing relationships between the size and frequency of large bursts in strains SX701 and SX703. Finally, we show that neither one of the two strains follow the negative binomial (or Gamma) distribution.

The paper is organized as follows. In the **Analysis** section, we describe the model, derive the master equation, and explain the key approximations used to obtain the steady state protein distribution. In the **Results** section, we perform simulations to check the validity of the analytical expression for the protein distribution, and we derive the expressions for mean and the variance of the distribution. We also show that the mean, variance, and hence, the Fano factor and the reciprocal of the noise, can be expressed in terms of the size and frequency of the transcriptional and translational bursts. In the **Discussion** section, the latter are compared with the assumptions of Choi et al. We also show that negative binomial distributions are obtained only if the size of the large transcriptional bursts is relatively small.

## Analysis

The model scheme, shown in Figure 1, is based on the following facts enunciated by Oehler et al. [25], [26]. The *lac* operon of *E. coli* contains three operators, namely the main operator 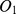, and the two auxiliary operators 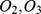, lying downstream and upstream of 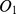. The *lac* operon rarely entertains more than one Lac repressor, and this single repressor 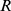 can bind to any one of the operators, thus forming the operon states, 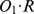, 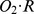, and 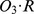. Since the tetrameric repressor is a "dimer of dimers,'' it has a free dimer even after it is bound to one of the operators. This free dimer can bind to one of the remaining two free operators, thus forming a DNA loop. In principle, three looped states are feasible, namely, 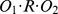, 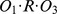, and 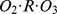, but the last one is very unlikely to form. We are therefore led to consider only six feasible states of the operon — the repressor-free state, and the five repressor-bound states, 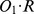, 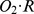, 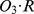, 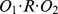, and 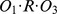. Only three of these six states permit transcriptional activity, namely, the repressor-free state and the repressor-bound states, 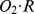 and 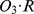. The first two states permit full transcriptional activity. The last state can be neglected since it permits only 3–5% of the full transcriptional activity.

The model kinetics are based on the following assumptions. All cells have the same number of repressors, 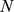, which is tantamount to neglecting extrinsic noise [4]. Since association of a cytosolic repressor to an operator is diffusion-limited, we assume that a cytosolic repressor has the same propensity, 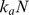, for association with each of the operators. In contrast, the propensity for dissociation of operator-bound repressor does depend on the identity of the operator, and we denote the propensity for dissociation of 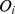 -bound repressor by 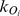. Next, we consider the kinetics of looping. The looped state 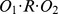 can be formed from either 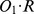 or 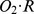, but both pathways have the same propensity because they are driven by the same local concentration effect [26]. Thus, we denote the propensity for formation of 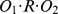 from 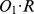 or 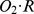 by the same symbol, 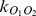. Similarly, we denote the propensity for formation of 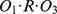 from 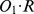 or 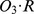 by the same symbol, 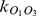. Finally, we let 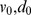 denote the propensities for mRNA synthesis and degradation, and 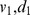 denote the propensities for protein synthesis and dilution.

### Equations

We take a master equation approach to describe the system, our state variables being the number of mRNAs, 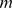, the number of proteins, 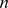, and the six states of the operon shown in Figure 1. We let 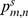 denote the probability of 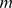 mRNAs and 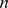 proteins when the operon is in state 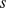. Here, 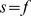 when the operon is free, and 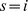 or 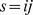 when the operon is repressor-bound, where 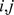 are integers identifying the operator(s) to which the repressor is bound (e.g., 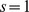 denotes the state 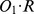 and 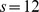 denotes the state 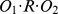). Then the master equations for the kinetic scheme in Figure 1 are
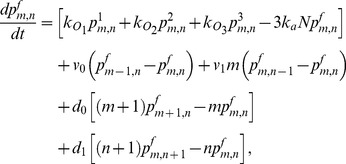
(1)




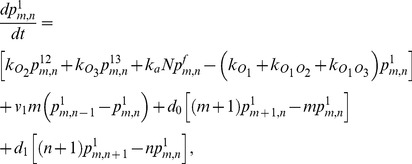
(2)




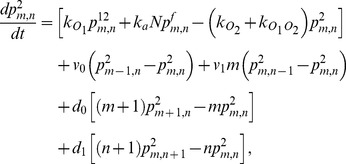
(3)




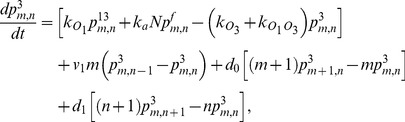
(4)




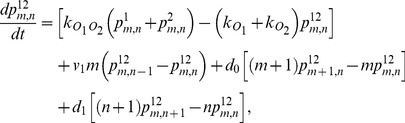
(5)




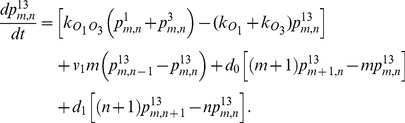
(6)


Our goal is to derive the steady state protein distribution corresponding to these equations.

### Parameter values

Table 1 shows the parameter values in the absence of the inducer. The parameters 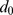 and 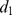 reflect the experimental values measured by Yu et al. [10]. The parameter 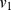 was chosen such that the the mean burst size, 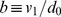, agreed with the measured value 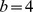, reported by Yu et al. The parameter 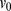 was estimated by assuming that the mean burst frequency of fully induced cells, 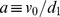, is 600. The rationale for this assumption is as follows. An uninduced cell contains, on average, 0.5 molecules of the tetrameric LacZ [9], and hence, is expected to contain 2 molecules of the monomeric LacY. Since the number of LacY and LacZ molecules increases 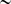
1200-fold in fully induced cells [25], there are 2400 LacY molecules in such cells, i.e., 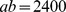, which implies that 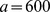. All other parameter values were estimated using the method of Vilar & Leibler [15]. They estimated all the equilibrium constants using the repression data of Oehler et al. [26]. Then, given an experimental estimate of any one parameter, they could find all other parameter values. They took that one parameter to be the dissociation rate constant, 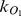, and assigned to it the value obtained from *in vitro* data [27]. Based on this procedure, the association rate, 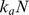, was found to be 0.73 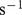. However, recent *in vivo* measurement show that the association rate for a dimeric repressor is 0.014 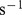 [28]. If the dimeric and tetrameric repressor associate at the same rate, and each cell contains 10 repressors [29], the estimated value of 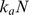 from these measurements is 0.14 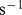. We assumed 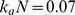 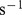, and chose 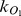, 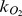, 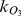, 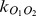, 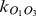 to ensure consistency with the repression data. As we show later, these parameter values yield good fits of the experimental data.

**Table 1:** Parameter values in the absence of inducer.

Parameter	Value (in 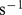 )	Parameter	Value (in 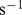 )
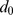	0.011	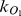	0.0016
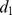	0.0002	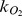	0.019
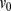	0.12	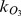	0.73
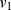	0.044	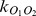	4
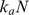	0.07	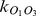	24

Since we are also concerned with protein distributions in the presence of the inducer, it is necessary to identify the parameters that change under these conditions. We assume that 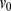, 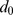, 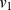, and 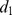 are independent of the inducer level. The propensities for looping, 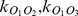 , are also unlikely to change in the presence of small inducer concentrations because a partially dissociated repressor has too little time to interact with the inducer: In the presence of 10* µ*M IPTG (considered equivalent to 100* µ*M TMG), the pseudo-first-order rate constant for repressor-inducer binding is 0.1 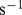 [30], which is negligible compared to the looping rate constant of 4 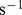. Thus, the only parameters that can change with the inducer concentration are the association rate, 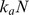, and the dissociation rates, 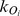. Based on the analysis of their experimental protein distributions, Choi et al. concluded that the dissociation rates are independent of the inducer concentration, while the association rate decreases with the inducer concentration. We shall also assume that this is the case. This assumption holds only if the concentration of TMG is significantly below 1 mM [31], [32], a condition satisfied by all the concentrations used by Choi et al., except possibly the highest concentration of 200* µ*M.

### Model reduction

The determination of the steady state protein distribution corresponding to eqs. (1)–(6) is facilitated by the fact that loop formation and mRNA degradation are relatively fast.

#### Rapid loop formation

Table 1 shows that in the absence of the inducer, 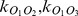 are much greater than all other propensities, and as explained above, this persists even in the presence of low inducer concentrations. It follows that the repressor-bound states rapidly equilibrate on the fast time scale 
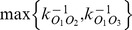
, after which there are relatively infrequent transitions between the repressor-free and repressor-bound states. To capture this physical fact, we replace eq. (2) with the equation for the slow variable
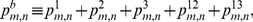
(7)which represents the probability of 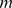 mRNAs and 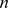 proteins when the operon is repressor-bound. We then apply the quasi-steady state approximation to the fast variables, 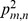, 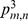, 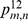, 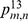, and find that the probabilities of the equilibrated bound states are given by the relations



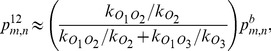
(8)

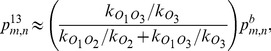
(9)




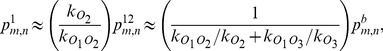
(10)




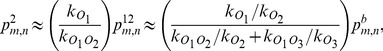
(11)




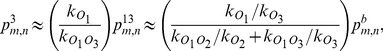
(12)which express the physical fact that after the bound states reach quasi-equilibrium, they obey the principle of detailed balance and are almost always in one of the looped states (Table 2). Moreover, the slow variables follow the equations

**Table 2:** Magnitudes of important derived parameters in the absence of the inducer.

Parameter	Value	Parameter	Value
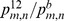	0.86	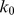	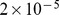 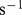
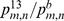	0.14	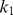	0.22 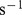
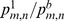	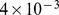	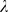	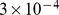
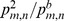	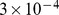	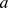	600
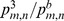	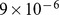	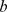	4



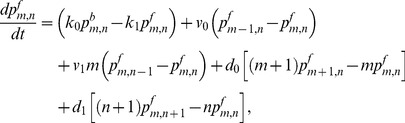
(13)




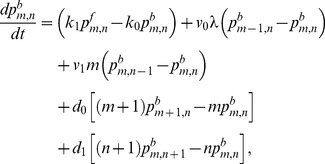
(14)where



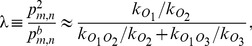
(15)

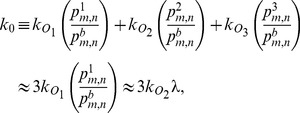
(16)




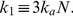
(17)


Equations (13)–(14) describe the evolution of the reduced model containing only two operon states — the free and the equilibrated bound states — between which are transitions with propensities, 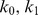, which are slow compared to the propensities for looping (Table 2). This is highlighted in Figure 1 by enclosing the free and bound states in dashed boxes, and drawing dashed arrows with labels, 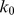 and 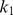, to denote the transitions between them. The reduced model is similar to Shahrezaei & Swain's three-stage model for a regulated promoter [22], but there is an important difference. Both operon states are transcriptionally active: The transcription rates in the free and bound states are 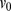 and 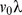, respectively, where 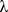 is the probability of the 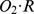 state. Even though 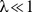 (Table 2), we cannot neglect the transcription from the bound state, since it captures the effect of the small transcriptional bursts, which can account, as we show later, for almost 80% of the mRNAs synthesized per cell cycle.

Table 2 shows that in the absence of the inducer, 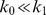, so that the free state occurs infrequently and lasts for very short periods of time, i.e., 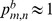. We shall show later that this persists in the presence of the low inducer concentrations (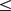 200* µ*M TMG) used by Choi et al. Hence, under the experimental conditions of interest, the conditional probabilities in (8)–(12) are essentially equal to the absolute probabilities.

#### Rapid mRNA degradation

The second approximation appeals to the fact that mRNA degradation is rapid compared to protein dilution, i.e., 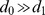. To apply this approximation, we follow Shahrezaei & Swain [22]. Thus, we begin by rescaling time with respect to the time scale for protein degradation. Letting 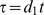 transforms the reduced equations to the form
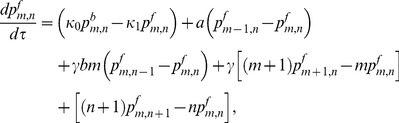
(18)




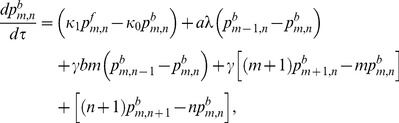
(19)where 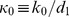 and 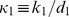 are the frequencies of transitions between the free and bound operator states, 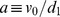 is the frequency of unregulated transcription (in the absence of the repressor), 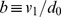 is the translational burst size, i.e., the average number of proteins produced per mRNA, and 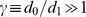 is the ratio of protein and mRNA lifetimes. Next, we define the generating functions, 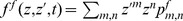 and 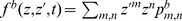, to obtain the partial differential equations



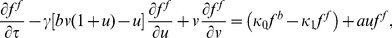
(20)

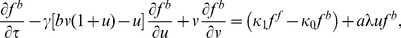
(21)where 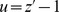 and 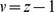. Since 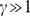, we have the quasi-steady state approximation, 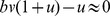. The steady state protein distribution is therefore given by the equations



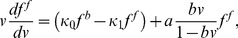
(22)

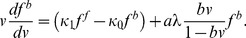
(23)


Since we are interested in the generating function, 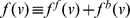, it is convenient to rewrite these equations as
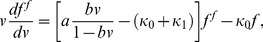
(24)




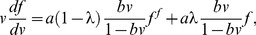
(25)which reduce to the second-order differential equation



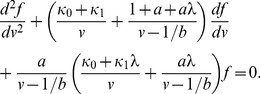
(26)We solve this equation with the initial condition, 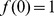, and revert to 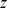 as the independent variable, to obtain the following generating function for the steady state protein distribution
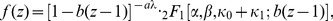
(27)where 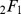 denotes the Gaussian hypergeometric function and



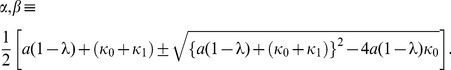
(28)As expected, if 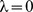, (27) reduces to the generating function of the negative hypergeometric distribution [22]. In general, however, (27) is the generating function for a mixture of the negative binomial and negative hypergeometric distributions, which reflects, as we show below, the existence of two sub-populations of proteins, namely those derived from small and large transcriptional bursts.

## Results

### Analytical expressions for the statistics of the protein distributions

#### Strain with auxiliary operators

The generating function (27) yields the following expressions for the mean, 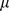, and variance, 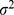, of the protein distribution
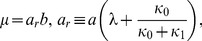
(29)




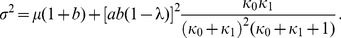
(30)


Since 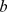 represents the mean number of proteins synthesized per mRNA, (29) implies that 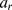 is the mean frequency of *regulated* transcription. The two terms of 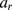 also have simple physical interpretations: Since 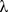 and 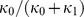 are the probabilities of the 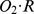 and free states, 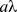 and 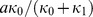 represent the mean number of mRNAs produced per cell cycle due to small and large transcriptional bursts.

Expanding 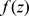 about 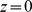 yields the steady state protein distribution
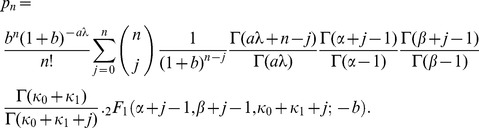
(31)


**Figure 3:**
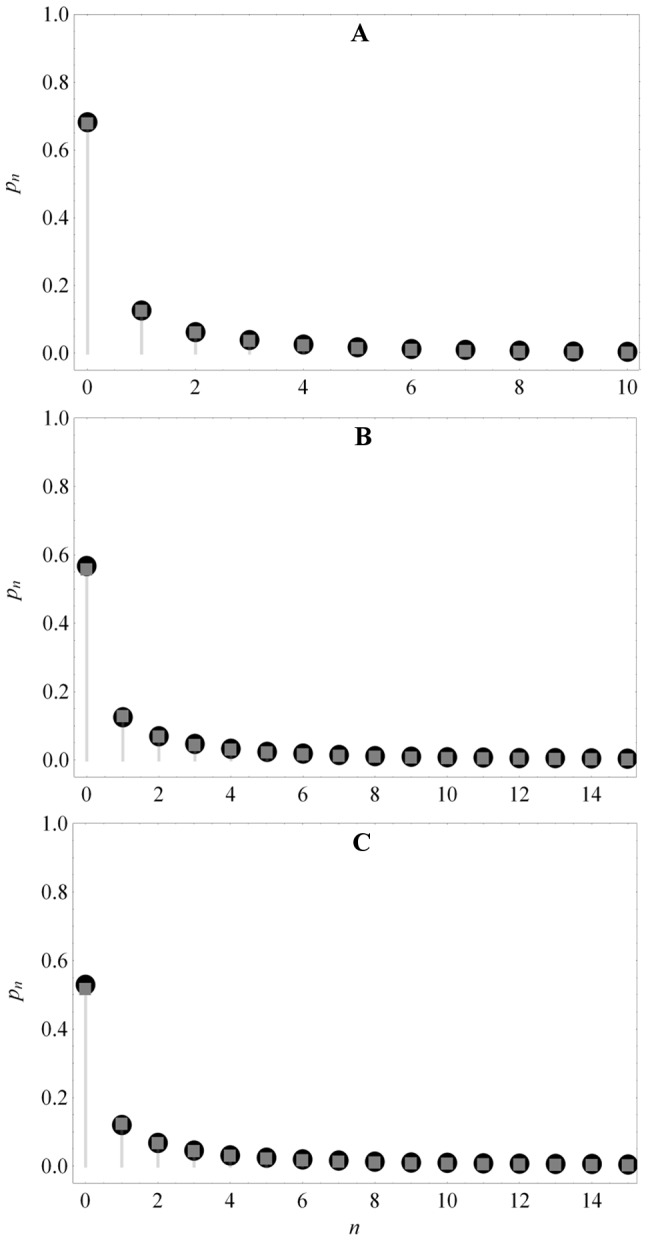
Despite a 20-fold change in the repressor association rate, 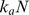, the protein distributions derived from the analytical expression (31) (grey squares) are in good agreement with those obtained from stochastic simulations of the model (black disks). (a) Parameter values in Table 1. (b) 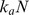 is 1/10th of the value in Table 1; other parameter values as in Table 1. (c) 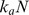 is 1/20th of the value in Table 1; other parameter values as in Table 1.

Figure 3 shows that the protein distributions obtained from this expression agree well with those obtained by simulating the full model with the Optimized Direct Method implementation of Gillespie's Stochastic Simulation Algorithm [33] provided in the simulation package StochKit2 [34]. The protein distribution in the absence of the inducer, shown in Fig. 3a, was obtained with the parameter values in Table 1. The distributions in the presence of the inducer were obtained by decreasing the association rate, 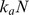, 10-fold (Fig. 3b) and 20-fold (Fig. 3c). Evidently, (31) is a good approximation to the exact solutions in all three cases. We conclude that our approximate solution is accurate down to a 20-fold reduction of the association rate.

Table 2 shows that in the absence of the inducer, 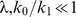. These relations remain valid at the relatively low inducer levels studied by Choi et al. (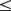
200* µ*M TMG). Indeed, under these conditions, the operon is expressed to no more than 1% of the fully induced level [12], i.e.,
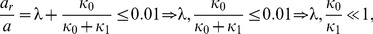
(32)and (29)–(30) can be rewritten as



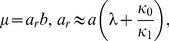
(33)

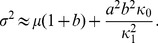
(34)


It is worth noting that due to rapid loop formation, small transcriptional bursts are very bursty (pulsatile). Moreover, under the weakly inducing conditions used in the experiments (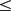 200* µ*M TMG), 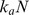 is relatively large, and hence, the large transcriptional bursts are also quite bursty. It follows that under these conditions, (33)–(34) should be expressible in terms of the size and frequency of the small and large transcriptional bursts. We shall show below that this is indeed the case.

#### Strain without auxiliary operators

In the absence of auxiliary operators, the operon fluctuates between the free and the 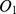-bound state, and only the former allows transcription. This is identical to Shahrezaei & Swain's 3-stage model of a regulated promoter [22], and corresponds to the special case, 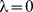, 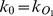, 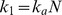 of our model. It follows that the generating function for the steady state protein distribution is the Gaussian hypergeometric function
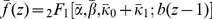
(35)where
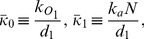
(36)and



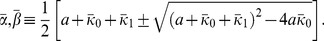
(37)Moreover, the protein distribution is given by the expression
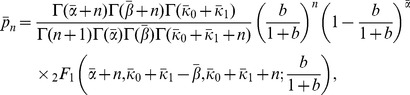
(38)and the mean and variance are



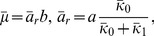
(39)

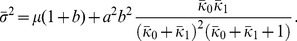
(40)


At TMG concentrations of 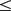 100* µ*M, which are equivalent to an IPTG concentration of 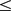 10* µ*M, the operon is expressed to no more than 5% of the fully induced level [35]. It follows that under the experimental conditions of interest
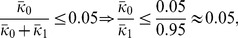
(41)and 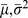 can be approximated by the expressions



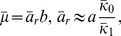
(42)

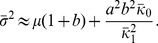
(43)


### Expressing the statistics in terms of the burst size and frequency

Choi et al. assumed that the quantities 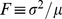 and 
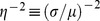
 represent the size and frequency of small transcriptional bursts, and 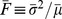 and 
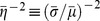
 represent the size and frequency of large transcriptional bursts. To check the validity of these assumptions, we shall express (33)–(34) and (42)–(43) in terms of the size and frequency of the transcriptional bursts. Given these expressions, we can immediately infer the dependence of 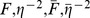 on the size and frequency of the transcriptional bursts, and then compare them to the assumptions made by Choi et al.

#### Strain with auxiliary operators

To express 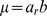 in terms of the size and frequency of the transcriptional bursts, we begin by recalling that 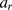 consists of two terms, 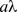 and 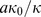, which represent the mean frequency of transcription due to partial and complete dissociations of the repressor, respectively. Since partial dissociations occur when a repressor trapped in the 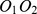 -loop dissociates from 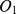, we define the number of the partial dissociations per cell cycle as
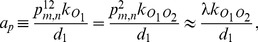
(44)where we have appealed to the detailed balance between the operon states 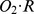 and 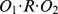. We also define the number of mRNAs synthesized per partial dissociation as
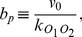
(45)since the time for rebinding of a partially dissociated repressor to 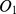 is on the order of  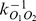. It follows from these definitions that
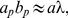
(46)i.e., we have successfully expressed the first term of 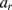 in terms of frequency and mRNA burst size due to partial dissociations. We now proceed to express the second term of 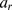 in terms of the frequency and mRNA burst size due to complete dissociations. Since complete dissociations occur whenever the operon becomes repressor-free, it is natural to define the number of complete dissociations per cell cycle as



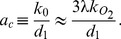
(47)We also define the number of mRNAs synthesized per complete dissociation as
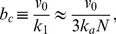
(48)because the time for rebinding of a completely dissociated repressor to an operator is on the order of 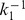. Evidently
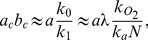
(49)and we conclude that



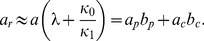
(50)Hence, (33)–(34) can be rewritten as
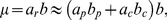
(51)




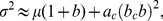
(52)which imply that



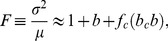
(53)

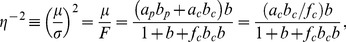
(54)where
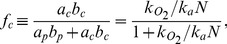
(55)is the fraction of proteins derived from complete dissociations. It follows from (53) that the total burstiness, 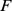, is entirely due to translational and large transcriptional bursts. Moreover, the burstiness of large transcriptional bursts depends on their intrinsic burstiness, 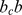, suitably weighted by 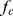, the fraction of proteins derived from such bursts. Importantly, 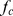 is completely determined by 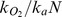, the equilibrium constant for dissociation of the repressor from 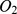. In the absence of the inducer, this equilibrium constant is 0.25 [25], [26], and hence, 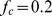, i.e., 20% of the proteins are derived from large transcriptional bursts. As the inducer concentration increases, 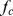 increases because 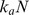 decreases.

#### Strain without auxiliary operators

In this case, if we define the number of complete dissociations per cell cycle as
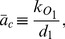
(56)and the number of mRNAs synthesized per complete dissociation as
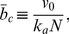
(57)the mean frequency of regulated transcription can be rewritten as



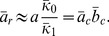
(58)It follows that (42)–(43) can be rewritten as
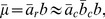
(59)




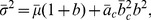
(60)which imply that



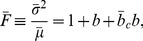
(61)

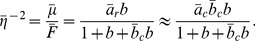
(62)


We are now ready to address questions concerning the physical meaning of the parameters of the distribution and their variation with inducer concentration [12].

## Discussion

### Interpretation of the protein distribution data

#### Strain with auxiliary operators. Interpretation of 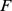 and 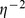 derived from filtered data

Choi et al. assumed that 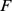 and 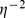 derived from the filtered data (Fig. 2c) represent the size and frequency of small transcriptional bursts. In terms of our model, these assumptions have the form
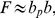
(63)

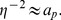
(64)


However, (53)–(54) imply that this 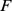 and 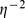, obtained by eliminating the contribution of the large transcriptional bursts, have a different physical meaning. Indeed, (53) implies that the Fano factor obtained from the filtered data has the form, 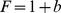, which represents the size of the translational, rather than small transcriptional, bursts. Similarly, (54) implies that the reciprocal of the noise derived from the filtered data has the form, 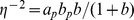, which is proportional to 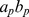, the average number of mRNAs derived from small bursts, rather than the frequency of the small bursts. Since 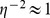 (Fig. 2c) and 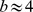, our interpretation of the filtered data implies that 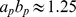, which is close to the estimate obtained from the model (Table 3).

**Table 3:** Burst frequency and size in uninduced cells with and without auxiliary operators.

Strain	With auxiliary operators						Without auxiliary operators		
Bursts due to	Partial dissociations			Complete dissociations			Complete dissociations		
Burst properties	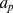	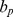	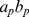	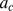	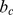	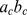	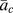	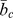	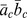
Model-based value 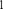	6.9	0.03	0.20	0.1	0.55	0.05	8	1.65	13
Data-based value 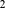	—	—	1.25	0.2			3		

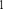
 Calculated from eqs. (44)–(45), (47)–(48), and (56)–(57) with the parameter values in Table 1.

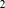
 Determined from the data in Figs. 2 a–c by appealing to eqs. (53)–(54) and (61)–(62).

Evidently, there is a discrepancy between the assumptions of Choi et al. and the implications of our model. To understand its origin, observe that their assumptions are equivalent to the relations
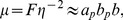
(65)




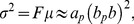
(66)i.e., they assumed, in effect, that both the mean and the variance are dominated by contributions from small transcriptional bursts. In contrast, (51)–(52) show that small bursts contribute to the mean, but not to the variance. This difference arises because we assumed that looping is so fast that the rapid fluctuations due to partial dissociations are averaged out on the slow time scale of the other processes. This averaging process preserves the contribution of small transcriptional bursts to the mean, but eliminates their contribution to the variance.

The assumption 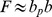 appears to be implausible. Indeed, (53) implies that translational bursts contribute the term 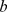 to the Fano factor. For the small bursts to make a significant, let alone dominant, contribution to the Fano factor, it is clear that 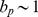, i.e., on average, approximately one mRNA must be synthesized per partial dissociation. However, looping is so fast compared to transcription that 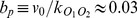 in the absence of the inducer (Table 3). Moreover, 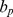 is unlikely to change even in the presence of the inducer since 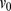 and 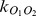 are constant over the range of inducer concentrations used in the experiments. We conclude that the bursts due to partial dissociations are so small that they cannot be the dominant source of burstiness.

#### Interpretation of 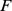 and 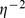 derived from raw data

Choi et al. rejected the raw data shown in Fig. 2b since the occurrence of large bursts in a few cells distorted the statistics of the small bursts. We show below that these data are a valuable source of information about the statistics of *large* bursts. Specifically, (53)–(54) predict the observed variation of 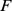 and 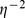 derived from the raw data, and thus provide a method for estimating not only the size and frequency of the large transcriptional bursts, but also the fraction of proteins derived from them. This method is particularly useful because, as we show below, there are simple relationships between the size and frequency of the large bursts in strains SX701 and SX703, but they are *not* identical.

The analysis of the raw data shows that the total burstiness, 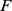, increases with inducer concentration (Fig. 2b). Eq. (53) implies that this is due to the growing burstiness of the large transcriptional bursts: Since both 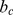 and 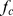 increase with inducer level, so does 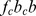. This increase occurs so rapidly that at 100* µ*M TMG, large trancriptional bursts become the dominant source of burstiness, i.e, 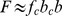. Indeed, assuming 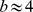, (53) implies 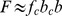 whenever 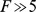. Inspection of Fig. 2b shows that at 100* µ*M TMG, 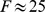, and hence, 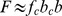. We shall show below that at such inducer levels, 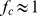 and 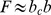.

In contrast to the total burstiness, 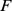, the reciprocal of the total noise, 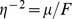, decreases with inducer concentration until it reaches a constant value (Fig. 2b). The model suggests that this is because both 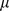 and 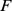 increase with inducer level, but 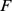 increases faster than 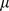: Indeed, both 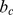 and 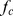 increase with inducer level, and Eq. (54) shows that 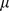 is proportional to the ratio 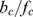, whereas 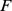 increases with the product 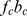. The decreasing trend of 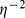 continues until the inducer levels become so high that large bursts account for all the proteins (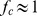) and burstiness (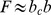). Under these conditions 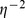 approaches 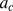, the frequency of large bursts, which is independent of inducer concentration. Comparison with the data in Fig. 2b then implies that 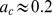.

Given 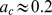 and 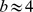, (53)–(54) provide a method for estimating the variation of 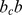 and 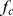 with inducer levels from the raw data for SX701. To see this, it is convenient to rewrite (53)–(54) in the form
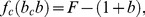
(67)




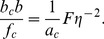
(68)


Since the variation of 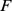 and 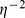 with the inducer concentration is known (Fig. 2b), we can solve the above equations to obtain 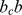 and 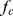 as a function of the inducer concentration. These calculated profiles, shown in Fig. 2d, agree with the claims above: Both 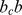 and 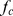 increase with the inducer level, and the latter approaches 1 at 100* µ*M TMG.

#### Strain without auxiliary operators. Interpretation of 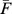 and 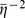


Choi et al. assumed that the 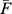 and 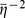 shown in Fig. 2a represent the size and frequency of large transcriptional bursts, i.e.,
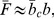
(69)




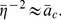
(70)


Our model implies that these relations are valid at all non-zero inducer concentrations used in the experiments. Indeed, since 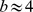, (61)–(62) imply that the above relations are valid whenever 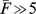, which is satisfied (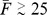) at all the non-zero inducer concentrations used in the experiments (Fig. 2a). In particular, comparison with the data in Fig. 2a implies that 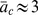.

#### Relationships between the statistics of large bursts in the strains with and without auxiliary operators

The model predicts simple relationships between the size and frequency of the large transcriptional bursts in strains SX701 and SX703, which provide tests for checking the consistency of the model. Indeed, it follows from (48) and (57) that 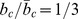, a relationship that is also mirrored by the data (compare full and dashed lines in Fig. 2d). Similarly, (47) and (56) imply that
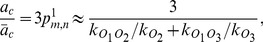
(71)a ratio estimated to be 1/80 based on the values in Table 1, which is of the same order of magnitude as the value 1/15, obtained from the experimentally determined values of 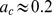 and 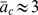.

### Condition for the negative binomial distribution

Choi et al. assumed that the protein distributions of both strains follow the Gamma distribution, the continuous analog of the negative binomial distribution. We have shown above that neither one of the strains follows the negative binomial distribution. Here, we demonstrate that the distributions can reduce to the negative binomial distribution, but only only if the large burst size is negligibly small, i.e., the association rate 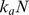, is much larger than the transcription rate 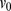. Under this condition, even the large bursts are averaged out, and they contribute to the mean, but not the variance or the burstiness.

We begin by considering the strain without auxiliary operators. Under the weakly induced conditions used in the experiments, 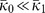, and the generating function for the protein distribution is the negative hypergeometric function
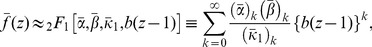
(72)which reduces to the generating function for the negative binomial distribution precisely when 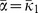 or 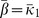. Now (37) implies that



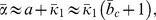
(73)

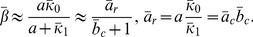
(74)


The condition 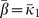 can never be satisfied since 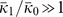. However, 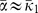 precisely when 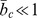, in which case 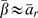 and
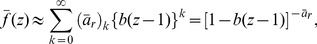
(75)which is the generating function for the negative binomial distribution



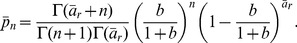
(76)It is worth noting that under this condition
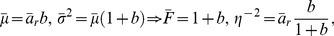
(77)i.e., large transcriptional bursts make no contribution to the burstiness.

A similar argument shows that the generating function for the strain with auxiliary operators reduces to
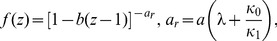
(78)precisely when 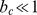. Under this condition, the proteins follow the negative binomial distribution



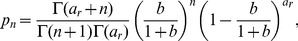
(79)and
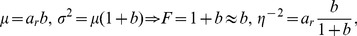
(80)i.e., even the large transcriptional bursts do not contribute to the burstiness.

**Figure 4:**
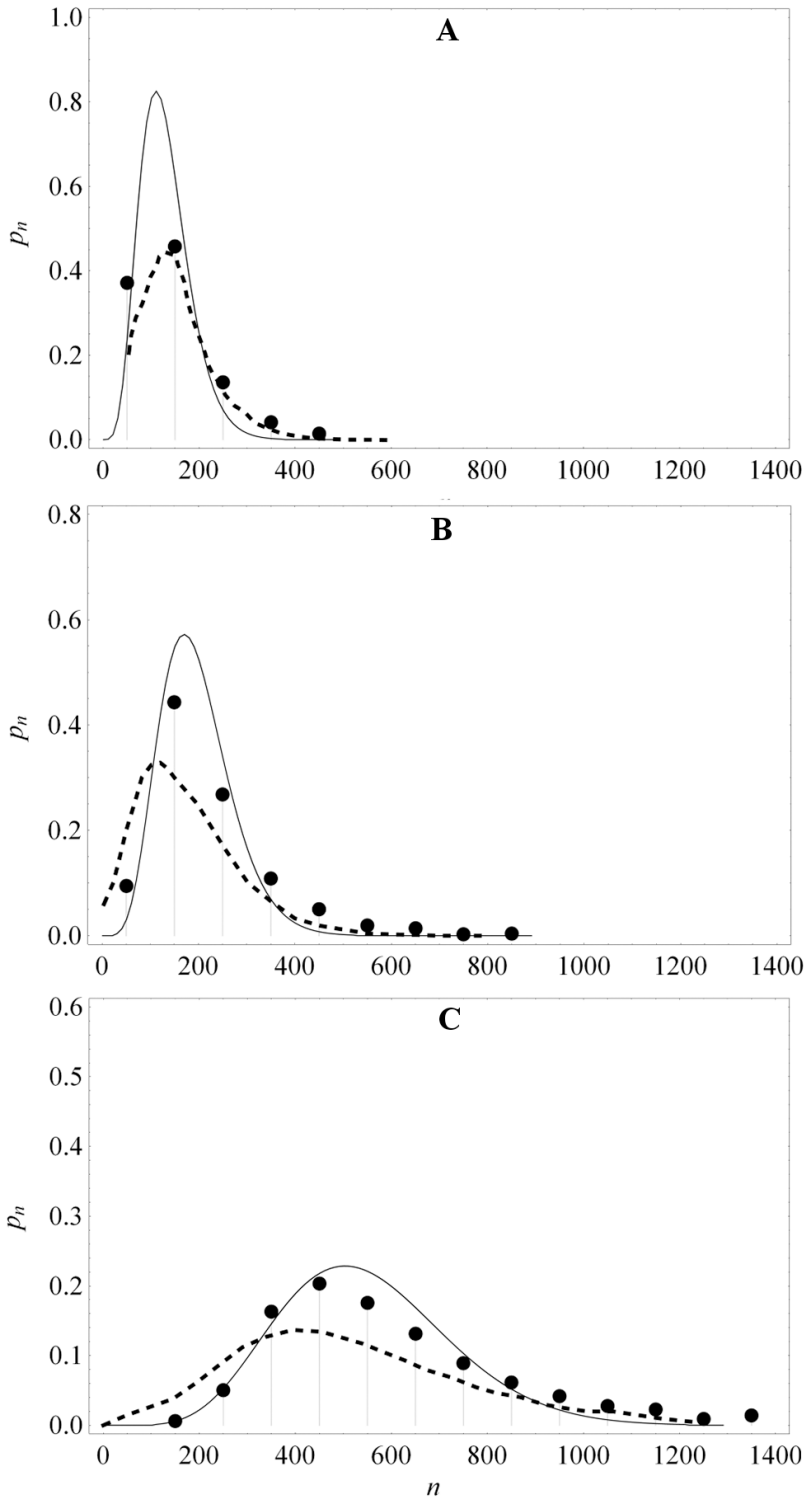
Protein distribution data for strain SX703 (full circles) at various TMG concentrations fitted with the Gamma distribution by Choi et al. (dashed curve) and the negative hypergeometric distribution (full curve). The negative hypergeometric distribution was fitted with the parameter values in Table 1, except 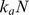, which was decreased with increasing inducer concentration. (a) Data obtained at 50* µ*M TMG fitted with 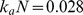 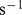. (b) Data obtained at 100* µ*M TMG fitted with 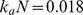 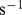. (c) Data obtained at 200* µ*M TMG fitted with 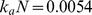 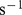.

We have shown above that the proteins follow the negative binomial distribution only if the large bursts are, in fact, rather small, and hence, do not contribute to the burstiness. But it follows from the data in Figs. 2a,b that these bursts do contribute significantly to the burstiness of strains SX701 and SX703 — if this was not true, (77) and (80) imply that the burstiness would be independent of inducer concentration, which contradicts the data. The negative binomial distribution is therefore unlikely to provide good fits to the raw data for both strains, but will fit the filtered data well, since the contribution of large bursts has been eliminated from it. The fits in Choi et al. are consistent with this conclusion. The Gamma distribution fits the filtered data for strain SX701 rather well. However, this is less so for the protein distributions obtained with strain SX703, which exhibits only large bursts. Figure 4 shows that better fits are obtained with the negative hypergeometric distribution (38).

## Conclusions

We formulated and solved a stochastic model of *lac* expression accounting for auxiliary operators and DNA looping. Based on a comparison of our expressions for the Fano factor, noise, and protein distribution of strains SX701 (with auxiliary operators) and SX703 (without auxiliary operators) with those proposed by Choi et al., we arrive at the following conclusions:

1. The physical interpretations of the Fano factor 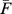 and reciprocal noise 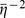 for strain SX703 are identical to those proposed by Choi et al., namely 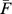 and 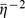 represent the size and frequency of (large) transcriptional bursts.
2. The physical interpretations of the Fano factor 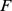 and reciprocal noise 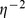 derived from the *filtered* data for SX701 differ from those given by Choi et al., namely 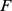 and 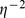 represent the size and frequency of small transcriptional bursts. Instead, we find that 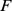 represents the size of translational bursts, and 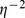 is proportional to the mean number of mRNAs derived from small transcriptional bursts. Our interpretation is different because we assume that looping is so fast that fluctuations due to small transcriptional bursts are averaged out — small bursts therefore contribute to the mean, but not the burstiness, of the protein distribution. This has two consequences:
  - The information lost due to the averaging implies that the small burst size and frequency cannot be separately extracted from the data. At best, we can only determine the product of the small burst size and frequency, which represents the mean number of mRNAs derived from small bursts.
  - The burstiness is entirely due to translational and large transcriptional bursts. In particular, the burst size derived from the filtered data for strain SX701, from which the contribution of the large bursts has been deliberately eliminated, yields the size of translational, rather than small transcriptional, bursts.
3. Choi et al. did not consider the *raw* data for SX701 because large bursts, although rare, contributed significantly to protein synthesis. This is consistent with our model: Even in uninduced cells, 20% of the proteins are derived from large bursts. We find that the raw data contains valuable information about the statistics of large bursts. By analyzing this data with our model, we isolate not only the size and frequency of large bursts, but also the fraction of proteins derived from them. The large burst size obtained in this manner is consistent with another prediction of the model, namely, it is one-third of the (large) burst size in strain SX703. The model also predicts that the fraction of proteins derived from large bursts is completely determined by a measurable quantity, namely the dissociation constant for binding of the repressor to the auxiliary operator 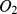.
4. The protein distributions for both strains are not negative binomial: SX703 follows a negative hypergeometric distribution, and SX701 follows a mixture of the negative binomial and negative hypergeometric distributions that reflects the existence of two sub-populations of proteins, namely, those derived from small and large bursts. Negative binomial distributions are attained only if large bursts are insignificant, a condition that holds only if the data are filtered by eliminating the contribution of such bursts.

These results imply that interpretation of the steady state protein distributions depends crucially on the details of the regulatory mechanisms.
